# The bereavement experience of adolescents and early young adults with cancer: Peer and parental loss due to death is associated with increased risk of adverse psychological outcomes

**DOI:** 10.1371/journal.pone.0181024

**Published:** 2017-08-23

**Authors:** Liza-Marie Johnson, Carlos Torres, April Sykes, Deborah V. Gibson, Justin N. Baker

**Affiliations:** 1 Division of Quality of Life and Palliative Care, St. Jude Children’s Research Hospital, Memphis, Tennessee, United States of America; 2 Department of Psychology, University of Memphis, Memphis, Tennessee, United States of America; 3 Department of Biostatistics, St. Jude Children’s Research Hospital, Memphis, Tennessee, United States of America; Neuroscience, SWEDEN

## Abstract

**Background:**

Adolescents commonly experience loss due to death, and perceived closeness to the deceased can often increase the intensity of bereavement. Adolescents and early young adult (AeYA) oncology patients may recall previous losses or experience new losses, possibly of other children with cancer, while coping with their own increased risk of mortality. The bereavement experiences of AeYA patients are not well described in the literature.

**Methods and findings:**

This analysis of bereavement sought to describe the prevalence and types of losses, the support following a death, and the impact of loss on AeYAs aged 13–21 years with malignant disease (or a hematologic disorder requiring allogeneic transplant). Participants were receiving active oncologic therapy or had completed therapy within the past 3 years. Participants completed a bereavement questionnaire and inventories on depression, anxiety, and somatization. The cross-sectional study enrolled 153 AeYAs (95% participation), most (88%) of whom had experienced a loss due to death. The most commonly reported losses were of a grandparent (58%) or friend (37%). Peer deaths were predominantly cancer related (66%). Many participants (39%) self-identified a loss as "very significant.” As loss significance increased, AeYAs were more likely to report that it had changed their life “a lot/enormously” (*P*<0.0001), that they were grieving “slowly or never got over it” (*P*<0.0001), and that they felt a need for more professional help (*P* = 0.026). Peer loss was associated with increased risk of adverse psychological outcomes (*P* = 0.029), as was parental loss (*P* = 0.018).

**Conclusions:**

Most AeYAs with serious illness experience the grief process as slow or ongoing. Peer or parental loss was associated with increased risk of negative mental health outcomes. Given the high prevalence of peer loss, screening for bereavement problems is warranted in AeYAs with cancer, and further research on grief and bereavement is needed in AeYAs with serious illness.

## Introduction

Adolescent loss due to death is surprisingly common; by the time they graduate from high school, most adolescents (71%) have experienced a loss, reporting a median of 2 deaths[1,2]. Most bereavement research in pediatrics has focused on children who have experienced the death of a parent or sibling due to illness or trauma [3–9], but peer and grandparent deaths are the losses most frequently reported by adolescents and early young adults (AeYAs)[2].

Bereavement is the objective situation of losing someone significant through death and the adjustment that follows, whereas grief refers to distress resulting from the loss and includes mental, physical, social or emotional difficulties[10]. An individual’s responses to bereavement may be influenced by many variables: their age and stage of development, their gender, a history of previous loss or trauma, the quality of their relationship with the deceased, their psychiatric history, and the type of loss (e.g., anticipated, traumatic, or violent)[10–12]. Violent or traumatic losses are associated with increased distress and maladaptive coping more often than are anticipated losses[13]. Loss due to death can be associated with physical and emotional health problems[14–16]. Somatic symptoms related to grief include headache, stomach pains, or gastrointestinal problems[17]. Adolescents may report feelings of shock, depression, loneliness, or anger; difficulty sleeping; feelings of emptiness, disbelief, hopelessness, or vulnerability; fear of intimacy; and sometimes guilt[1,15,16,18]. The loss may be associated with depressive symptoms or increased rates of major depression, or it may produce high levels of death anxiety and intense grief[2,19]. The loss of a friend within the past year may result in substance misuse or dependence[16].

Despite these potential difficulties arising from loss, adults in an adolescent’s life may not recognize the impact of loss, especially peer loss, and may, therefore, fail to provide adequate social support[1]. Parents who listen to their children’s concerns rather than ignore the loss event, or who offer advice, are considered helpful, and social support from peers is most helpful across all loss situations[1]. Teenagers with serious illness, such as cancer, often have access to additional support services (i.e., child life specialists, hospital chaplains, or social workers) that may serve as a source of support in recovering from a loss; however, it is unknown whether teenagers use these services to talk about loss.

Although AeYAs continue to rely on their parents for some social support, adolescence is also a period of increasing independence and the establishment of self with separation from the family and greater emphasis on peer relations. A number of developmental milestones characterize this period, including the ability to generalize, reason deductively, and deal with abstract ideas. Serious illness can make an AeYA “different” from their peers as they cope with treatment or disease effects, and it may complicate the achievement of developmental norms[20]. It is unknown if AeYAs exposed to loss in these circumstances use their parents or others as sources of social support.

In oncology, adolescents and young adults are commonly defined as individuals aged 13 to 39 years[21]; here, however, we will focus on the subset of AeYAs, who are 13 to 21 years of age. Individuals in this age group represent a distinct patient population because of their unique developmental status, one that is positioned between childhood and burgeoning adulthood. AeYAs in this age range undergo rapid brain maturation that results in neurocognitive advances such as the development of high-order thinking skills and the establishment of behavioral patterns[22]. This age group is also most representative of the AYA patients typically followed in a pediatric oncology center.

The overall 5-year survival rate for children with cancer has increased to roughly 85%[23], yet malignant disease remains the fourth leading cause of death (after accidental death, homicide, and suicide) in children through the age of 19 years[24]. Although children undergoing therapy for malignant disease will probably be aware of other children who die as a result of their illness, the frequency of friend loss is not well described among AeYAs with illness. There is a void in our understanding of how children with cancer process and cope with loss in their lives in the context of considering their own mortality due to illness.

Most AeYAs have developed the psychological maturity to realize that death is irreversible, and in order to minimize dissonance (between their feeling invincible and the reality of mortality), they may keep thoughts of dying at a distance[19,25]. Death anxiety occurs when the loss of another is a reminder of one’s own mortality, and this may subsequently trigger concerns about one’s personal potential for dying[19,26]. There is a time after loss when adolescents are “consumed by their irrevocably changed reality.”[27] In young adults (20–40 years of age) with advanced cancer, higher levels of grief due to cancer-related losses were associated with greater perceived life disruption, but the impact of this type of loss on younger AeYAs with cancer is unknown[28].

Because evidenced-based studies on grief and bereavement in AeYA populations are limited, particularly in the context of serious illness, the purpose of this study was to determine the prevalence of loss and the types of losses in AeYAs with serious illness (cancer) and to describe the general loss experience, with a focus on peer loss.

## Methods

This was a cross-sectional study in which the primary objective was to describe the prevalence, types, and importance of loss experienced by AeYA patients with a diagnosis of malignant disease or severe hematologic disorder requiring treatment by allogeneic stem cell transplant (i.e., severe aplastic anemia), along with the sources of support used by these patients after experiencing loss. Eligible AeYAs were receiving active therapy (they were eligible after 6 months of therapy or after 3 months if experiencing progressive disease) or had completed therapy within the previous 3 years. As an exploratory objective, participants were screened for risk of depression, anxiety, or somatization to determine whether there was a relation with bereavement. This single-institution study was approved by the Institutional Review Board at St. Jude Children’s Research Hospital; written assent and parental consent were obtained from every study participant in accordance with the institutional protocol.

### Participants and study procedures

**Fig 1:**
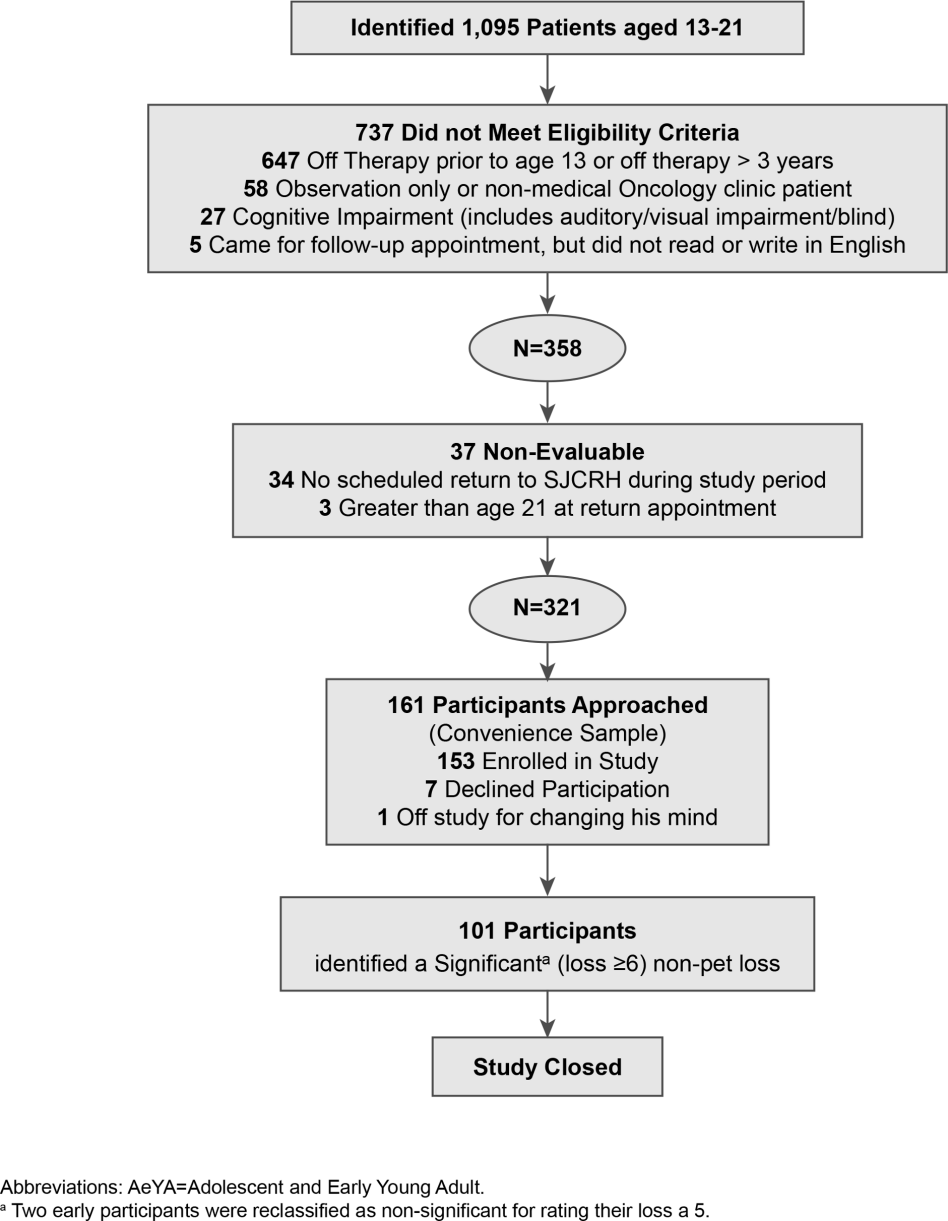
Screening and enrollment of AeYA particpiants.

In March 2012, by reviewing bioinformatics data, we identified 1095 patients at St. Jude Children’s Research Hospital who were 13 to 21 years of age and had a diagnosis of malignant disease or serious hematologic disorder requiring allogeneic bone marrow transplant. Patients were excluded if they had completed oncologic therapy before the age of 13 years. We excluded 737 individuals who failed to meet the inclusion criteria, and 37 patients were non-evaluable, leaving 321 potentially eligible participants (Fig 1). Enrollment was conducted through a convenience sample as eligible AeYAs were identified from a review of hospital scheduling records and approached during outpatient clinic visits or during routine hospital admission (i.e., for planned chemotherapy). Participants completed written surveys in the presence of a study team member, who was available to answer any questions or concerns that arose during survey completion.

### Study measurements

#### Bereavement questionnaire

Information on AeYA exposure to loss was collected using the bereavement questionnaire (BQ) of Harrison and Harrington, which was modified slightly for use in our population[2]. The modified BQ added questions about attendance at memorial services, the manner of death (expected, unexpected, traumatic, etc.), and whether the death was cancer related, S1 File. Those participants who identified any loss, including pet loss, were asked about the loss significance, the bereavement experience, and the sources of social of support they used after the death. Participants with multiple losses were asked to identify, if possible, their most important loss and to consider this loss when answering these questions. Losses occurring before the birth of the patient were excluded. Parental losses included step-parents, and grandparent losses included great-grandparents or step-grandparents. Second-degree relatives were defined as aunts, uncles, cousins, nieces, and nephews. Participants were asked to identify the significance of an identified loss (or of the most important loss, if there had been multiple losses) by using a 10-point Likert-based scale (whereby a rating of 1 corresponded to “not at all important,” a rating of 5 was “neutral,” and a rating of 10 corresponded to “very important”). For analysis, responses were grouped as having low importance (1–5), medium importance (6–8), or high importance (9–10). A rating of 5 was grouped with “low importance” because it encompassed “neutral” on the 10-point Likert scale.

#### Psychosocial distress

Depression (12 items), anxiety (13 items), and somatization (7 items) were assessed using the adolescent self-report subscales of the Behavioral Assessment System for Children (BASC-2) and examined for a relation to loss. The BASC-2 is a standardized assessment tool widely used to evaluate the behavior and emotions of individuals aged 2 to 25 years. The forms specifically for use with adolescents and young adults aged 12 to 21 years feature adequate psychometric properties, including sufficient reliability (with α’s ranging around 0.80) and validity[29]. Raw subscale scores were calculated by summing the scores for each item (using the standard approach specified by the developers of the instrument) and converted to clinical T-scores by using normed data based on age level. An individual with a T-score of 60 to 69 is considered at risk, and a score of 70 or higher is considered clinically significant[29]. For all analyses, depression, anxiety, and somatization T-scores were considered as 1) individual binary variables, using a cut point of a score of 60, and 2) a composite binary variable (yes/no), defined as a T-score of 60 or higher on at least one of the scales for depression, anxiety, or somatization. T-scores were considered as categorized values because they classify the population into those at risk (T-scores≥60) and those not at risk (T-scores<60) for psychological distress, which appears to be a clinically relevant and informative distinction. The importance of loss was categorized as follows for all analyses: low importance (1–5), medium importance (6–8), or high importance (9–10). Participants with clinically significant scores or scores indicative of being at risk for depression or anxiety were notified of their positive screening results and offered referrals to psychologists.

### Statistical analysis

Descriptive statistics (i.e., the number and percentage, mean and standard deviation, or median and range) were reported as appropriate. Fisher’s exact tests were used to examine the association between the type of loss and depression, anxiety, and somatization T-scores. Fisher’s exact tests or Mantel-Haenszel chi-square tests (with exact *P*-values reported) were used to compare the categorized age at enrollment, gender, race, cancer diagnosis, frequency of talking about loss, and depression, anxiety, and somatization T-scores across levels of loss importance. Kruskal-Wallis tests were used to compare discrete values of age at diagnosis and enrollment across levels of loss importance.

Statistical analyses were performed using SAS 9.3 software (SAS Institute, Cary, NC). All statistical tests were two sided, and *P*-values of less than 0.05 were considered statistically significant.

## Results

Of 161 AeYA patients invited to participate in the study, 153 (95%) were enrolled (Fig 1). The demographic and disease characteristics of the AeYA participants are listed in Table 1. Eighteen participants (12%) reported having experienced no losses, and 19 (12%) reported a pet loss as their only (n = 12, 7%) or most important loss. Multiple losses were common (being reported by 67% of participants), even when pet losses were excluded from the analysis (leaving 61% of participants having experienced multiple losses). The median number of losses was three per participant, with a range from one to eight. Excluding pet loss, the median number of losses fell to two per participant. The most common losses reported by AeYA oncology patients were a grandparent (58%), a friend (37%), a treasured pet (35%), or a second-degree relative (34%). Fifty-six participants (37%) reported a total of 95 friend (peer) losses, with 66% (n = 63) of these losses being cancer-related deaths. Table 2 shows the most important losses and their distribution across family relationships based on the level of loss importance.

**Table 1:** Demographic and disease characteristics by importance of loss.

			Of those reporting a “most important” loss (*N* = 114)			
		All participants (*N* = 153)	Importance of loss 1–5 (*N* = 14)	Importance of loss 6–8 (*N* = 41)	Importance of loss 9–10 (*N* = 59)	*P* ^b^
**Age at enrollment (years)**						
	13–18	101 (66%)	9 (64%)	28 (68%)	37 (63%)	0.889
	19–21	52 (34%)	5 (36%)	13 (32%)	22 (37%)	
	Mean (SD)	17.9 (2.2)	17.8 (1.9)	17.7 (2.2)	18.2 (2.1)	0.514
	Median (range)	18.0 (13.3–21.9)	18.0 (14.6–21.6)	17.7 (14.1–21.7)	18.1 (13.3–21.9)	
**Age at diagnosis (years)**						
	Mean (SD)	15.2 (2.6)	15.5 (1.4)	14.5 (2.7)	15.8 (2.2)	0.051
	Median (range)	15.4 (4.7–20.9)	15.7 (13.2–18.1)	14.2 (8.9–20.6)	15.8 (11.3–20.5)	
**Gender**						
	Male	86 (56%)	6 (43%)	25 (61%)	30 (51%)	0.464
	Female	67 (44%)	8 (57%)	16 (39%)	29 (49%)	
**Race**						
	White	115 (75%)	11 (79%)	33 (80%)	40 (68%)	0.629
	Black	29 (19%)	3 (21%)	5 (12%)	15 (25%)	
	Multiple race	5 (3%)	0 (0%)	1 (2%)	2 (3%)	
	Other	3 (2%)	0 (0%)	2 (5%)	1 (2%)	
	Unknown	1 (1%)	0 (0%)	0 (0%)	1 (2%)	
**Cancer diagnosis^a^**						
	Leukemias, myeloproliferative diseases, and myelodysplastic diseases	42 (27)	4 (29%)	10 (24%)	12 (20%)	0.98
	Lymphomas and reticuloendothelial neoplasms	36 (24)	3 (21%)	10 (24%)	15 (25%)	
	CNS and miscellaneous intracranial and intraspinal neoplasms	21 (14)	3 (21%)	4 (10%)	9 (15%)	
	Neuroblastoma and other peripheral nervous cell tumors	1 (1)	0 (0%)	1 (2%)	0 (0%)	
	Malignant bone tumors	22 (14)	2 (14%)	7 (17%)	10 (17%)	
	Soft tissue and other extraosseous sarcomas	12 (8)	1 (7%)	3 (7%)	8 (14%)	
	Germ cell tumors, trophoblastic tumors, and neoplasms of gonads	3 (2)	0 (0%)	0 (0%)	0 (0%)	
	Other malignant epithelial neoplasms and malignant melanomas	12 (8)	1 (7%)	4 (10%)	4 (7%)	
	Serious blood disorder requiring allogeneic transplant	4 (3)	0 (0%)	2 (5%)	1 (2%)	

Abbreviations: SD, standard deviation. NOTE: Participants who reported a pet as their only loss (or most important loss) were excluded from this analysis. A participant may have indicated more than one important loss.

^a^Categorized by the International Classification of Childhood Cancer, Third Edition (ICCC-3).

^b^Comparisons exclude the “unknown” category.

**Table 2:** Important losses reported by adolescents.

		“Most important” loss?		Of those identifying a “most important loss”		
Type of loss	All*	No	Yes	Importance of loss 1–5	Importance of loss 6–8	Importance of loss 9–10
Sibling(s)	7 (5%)	0 (0%)	7 (100%)	0 (0%)	2 (29%)	5 (71%)
Parent(s)	8 (5%)	2 (25%)	6 (75%)	0 (0%)	0 (0%)	6 (100%)
Grandparent(s)	89 (58%)	28 (31%)	61 (69%)	10 (16%)	22 (36%)	29 (48%)
Friend(s)	56 (37%)	27 (48%)	29 (52%)	2 (7%)	11 (38%)	16 (55%)
2^nd^-degree relative(s)	52 (34%)	31 (60%)	21 (40%)	2 (10%)	8 (38%)	11 (52%)
Treasured pet(s)	53 (35%)	32 (60%)	21 (40%)	9 (43%)	5 (24%)	7 (33%)
Other loved one(s)	8 (5%)	7 (88%)	1 (13%)	0 (0%)	1 (100%)	0 (0%)

NOTE: Reported loss occurred after the participant’s birth. A participant may have indicated more than one most important loss.

*Percentages reported are based on N = 153.

The study population identified 349 non-pet losses, and cancer-related deaths accounted for 38% of all losses, S1 Table. Seventeen percent of losses were traumatic (resulting from homicide, suicide, accidental gun death, or motor vehicle accident). The remainder resulted from natural causes (38%) or unknown causes (7%). Funeral or memorial services were attended for only 54% of the losses. Some participants noted being unable to attend services for a peer because of their own cancer-related therapy, and some regretted missing the event (field notations).

Table 3 shows the effect of the loss on the AeYA’s life, their recovery from the loss, and their need for more professional help. As the level of significance of the loss increased, the loss was more frequently discussed with others, was more likely to change the participant’s life, and was more likely to be difficult to get over (*P* = 0.001, <0.0001, and <0.0001, respectively). Participants identifying pet loss as their only or most important loss were excluded, leaving 114 evaluable AeYAs after two participants were excluded for failing to self-identify the importance of their loss on the Likert-based scale.

**Table 3:** Significance and impact of loss reported by adolescents.

		Of those reporting a “most important” loss, n (%)			
		Importance of loss 1–5	Importance of loss 6–8	Importance of loss 9–10	*P*
**The loss(es) changed my life…**					
	A little / It didn't change my life much	13 (93%)	33 (80%)	19 (32%)	**<0.0001^a^**
	A lot / Enormously	1 (7%)	8 (20%)	40 (68%)	
**I got over the loss(es)…**					
	Quickly / Right away	12 (86%)	17 (41%)	14 (24%)	**<0.0001^a^**
	Slowly / I never got over it	2 (14%)	24 (59%)	45 (76%)	
**Since the death(s) I have talked about the loss(es)…**					
	Rarely / Never	9 (64%)	21 (51%)	19 (32%)	**0.001^a^**
	Sometimes	5 (36%)	17 (41%)	25 (42%)	
	Often / Always	0 (0%)	3 (7%)	15 (25%)	
**I felt I needed more professional help…**					
	Rarely / Never	14 (100%)	38 (93%)	48 (81%)	**0.026^a^**
	Sometimes	0 (0%)	3 (7%)	10 (17%)	
	Often / Always	0 (0%)	0 (0%)	1 (2%)	

NOTE: Participants who reported a pet as their only loss (or most important loss) were excluded from the analysis. A participant may have indicated more than one important loss.

^a^Statistically significant, P<0.05.

Psychological determinants of distress (depression, anxiety, and somatization) are reported in Table 4. The loss of a parent was associated with being more likely to have a T-score of 60 or more on any of the BASC-2 subscales (i.e., for depression, anxiety, and/or somatization; *P* = 0.018). Risk for depression, anxiety, and/or somatization was also associated with loss of a friend (*P* = 0.029). Sibling losses did not negatively affect BASC-2 scores (*P* = 0.668).

**Table 4:** Important losses reported by adolescents.

		Any BASC-2			Depression			Anxiety			Somatization		
		T-score ≥60^a^			T-score			T-score			T-score		
Type of loss		No	Yes	*P*	<60	≥60	*P*	<60	≥60	P	<60	≥60	*P*
**Parent(s)**													
	No loss	114 (79%)	31 (21%)	**0.0180.015^b^**	135 (93%)	10 (7%)	**0.0210.015^b^**	124 (86%)	21 (14%)	**0.0250.015^b^**	130 (90%)	15 (10%)	0.218
	Loss	3 (38%)	5 (63%)		5 (63%)	3 (38%)		4 (50%)	4 (50%)		6 (75%)	2 (25%)	
**Sibling(s)**													
	No loss	112 (77%)	34 (23%)	0.668	134 (92%)	12 (8%)	0.47	123 (84%)	23 (16%)	0.321	129 (88%)	17 (12%)	1
	Loss	5 (71%)	2 (29%)		6 (86%)	1 (14%)		5 (71%)	2 (29%)	0.321	7 (100%)	0 (0%)	
**Grandparent(s)**													
	No loss	48 (75%)	16 (25%)	0.847	57 (89%)	7 (11%)	0.39	54 (84%)	10 (16%)	1	56 (88%)	8 (13%)	0.795
	Loss	69 (78%)	20 (22%)		83 (93%)	6 (7%)		74 (83%)	15 (17%)		80 (90%)	9 (10%)	
**2nd degree relative(s)**													
	No loss	81 (80%)	20 (20%)	0.16	94 (93%)	7 (7%)	0.367	88 (87%)	13 (13%)	0.113	93 (92%)	8 (8%)	0.104
	Loss	36 (69%)	16 (31%)		46 (88%)	6 (12%)		40 (77%)	12 (23%)		43 (83%)	9 (17%)	
**Friend(s)**													
	No loss	80 (82%)	17 (18%)	**0.0290.015^b^**	92 (95%)	5 (5%)	0.07	84 (87%)	13 (13%)	0.256	91 (94%)	6 (6%)	**0.015^b^**
	Loss	37 (66%)	19 (34%)		48 (86%)	8 (14%)		44 (79%)	12 (21%)		45 (80%)	11 (20%)	
**Other loved one(s)**													
	No loss	113 (78%)	32 (22%)	0.089	133 (92%)	12 (8%)	0.517	122 (84%)	23 (16%)	0.618	131 (90%)	14 (10%)	**0.045^b^**
	Loss	4 (50%)	4 (50%)		7 (88%)	1 (13%)		6 (75%)	2 (25%)		5 (63%)	3 (38%)	
**Pet(s)**													
	No loss	77 (77%)	23 (23%)	0.843	90 (90%)	10 (10%)	0.544	85 (85%)	15 (15%)	0.646	87 (87%)	13 (13%)	0.42
	Loss	40 (75%)	13 (25%)		50 (94%)	3 (6%)		43 (81%)	10 (19%)		49 (92%)	4 (8%)	
**Overall**													
	No loss	16 (89%)	2 (11%)	0.245	18 (100%)	0 (0%)	0.366	17 (94%)	1 (6%)	0.31	17 (94%)	1 (6%)	0.695
	Any type of loss	101 (75%)	34 (25%)		122 (90%)	13 (10%)		111 (82%)	24 (18%)		119 (88%)	16 (12%)	

^a^ Depression, Anxiety, and/or Somatization T-score ≥60.

^b^ Statistically significant, p<0.05.

AeYA participants were asked how frequently they talked about the death and with whom they talked about it (Table 5). As the level of loss significance increased, AeYAs talked more frequently about the losses with other adults in their family (*P* = 0.009); siblings (*P* = 0.008); a child life specialist, chaplain, or social worker (*P* = 0.014); or a mental health professional (*P* = 0.010). One third of participants rarely or never talked about their loss with anyone; although this was less likely to be the case as the level of loss significance increased (*P* = 0.002). Scores on the BASC-2 subscales did not vary across the level of loss significance. (*P* = 0.457 for any T-score ≥60) (Table 6).

**Table 5:** Who adolescents talked with about their loss.

Since the death(s) I have talked about it with…		Of those reporting a “most important” loss			
		Importance of loss 1–5	Importance of loss 6–8	Importance of loss 9–10	*P*
**Parents**					
	Rarely/Never	9 (64%	19 (46%)	27 (46%)	0.115
	Sometimes	5 (36%)	15 (37%)	15 (25%)	
	Often/Always	0 (0%)	7 (17%)	17 (29%)	
**Other adults in my family**					
	Rarely/Never	11 (85%)	26 (67%)	29 (50%)	**0.009^b^**
	Sometimes	2 (15%)	10 (26%)	20 (34%)	
	Often/Always	0 (0%)	3 (8%)	9 (16%)	
**Siblings**					
	Rarely/Never	10 (91%)	27 (68%)	28 (50%)	**0.008^b^**
	Sometimes	1 (9%)	9 (23%)	19 (34%)	
	Often/Always	0 (0%)	4 (10%)	9 (16%)	
**Friends**					
	Rarely/Never	13 (93%)	28 (68%)	35 (59%)	**0.037^b^**
	Sometimes	0 (0%)	10 (24%)	16 (27%)	
	Often/Always	1 (7%)	3 (7%)	8 (14%)	
**School counselor**					
	Rarely/Never	14 (100%)	40 (98%)	53 (95%)	0.454
	Sometimes	0 (0%)	1 (2%)	3 (5%)	
	Often/Always	0 (0%)	0 (0%)	0 (0%)	
**Doctor or nurse**					
	Rarely/Never	14 (100%)	39 (95%)	51 (86%)	0.057
	Sometimes	0 (0%)	2 (5%)	6 (10%)	
	Often/Always	0 (0%)	0 (0%)	2 (3%)	
**Bereavement counselor, child life specialist, chaplain or social worker**					
	Rarely/Never	14 (100%)	41 (100%)	52 (88%)	**0.014^b^**
	Sometimes	0 (0%)	0 (0%)	4 (7%)	
	Often/Always	0 (0%)	0 (0%)	3 (5%)	
**Child psychologist/therapist/psychiatrist**					
	Rarely/Never	14 (100%)	38 (93%)	47 (80%)	**0.010^b^**
	Sometimes	0 (0%)	3 (7%)	4 (7%)	
	Often/Always	0 (0%)	0 (0%)	8 (14%)	
**Online (social networking site, chat groups, online support groups)**					
	Rarely/Never	11 (92%)	36 (97%)	53 (91%)	0.467
	Sometimes	1 (8%)	0 (0%)	3 (5%)	
	Often/Always	0 (0%)	1 (3%)	2 (3%)	
**None of the above^a^**					
	No	5 (36%)	25 (61%)	46 (78%)	**0.002^b^**
	Yes	9 (64%)	16 (39%)	13 (22%)	

NOTE: Participants who reported a pet as the only loss (or most important loss) were excluded from the analysis. A participant may have indicated more than one important loss. The total N may not sum to 114 participants due to missing data.

^a^Indicated not applicable, never, or rarely to all questions.

^b^Statistically significant, p<0.05.

**Table 6:** Psychological outcomes and importance of loss.

				Of those reporting a “most important” loss						
		All participants (*N* = 153)		Importance of loss 1–5 (*N* = 14)		Importance of loss 6–8 (*N* = 41)		Importance of loss 9–10 (*N* = 59)		
BASC-2										
Depression T-score										*P*
	<60, n (%)	140 (92%)		13 (93%)		38 (93%)		51 (86%)		0.339
	≥60 n (%)	13 (8%)		1 (7%)		3 (7%)		8 (14%)		
Anxiety T-score										
	<60, n (%)	128 (84%)		11 (79%)		36 (88%)		46 (78%)		0.473
	≥60, n (%)	25 (16%)		3 (21%)		5 (12%)		13 (22%)		
Somatization T-score										
	<60, n (%)	136 (89%)		12 (86%)		38 (93%)		51 (86%)		0.626
	≥60, n (%)	17 (11%)		2 (14%)		3 (7%)		8 (14%)		
Any T-score ≥ 60^a^										
	No, n (%)	117 (76%)		10 (71%)		34 (83%)		42 (71%)		0.457
	Yes, n (%)	36 (24%)		4 (29%)		7 (17%)		17 (29%)		

Abbreviations: SD, standard deviation; BASC-2, Behavior Assessment System for Children, Second Edition. T-scores ≥ 60 on the BASC-2 reflect at-risk or clinically significant scores. NOTE: Participants who reported a pet as their only important loss were excluded. A participant may have indicated more than one important loss.

^a^Depression, anxiety, and/or somatization T-score ≥ 60.

## Discussion

Adolescent and early young adult patients with serious illness are likely to have experienced the loss of a family member or friend (n = 123, 80.4%). Not surprisingly, peer loss appears to be more common among these patients than in the general population of adolescents (being reported by 37% of patients versus 10.8% of the general population)[2], and two-thirds of peer losses are cancer related. Furthermore, it appears that having experienced the loss of a parent or peer, in the context of a cancer diagnosis, is associated with an increased risk of negative psychological outcomes in this population. We were surprised to find no relation between sibling loss and negative psychological outcomes, but the small number of sibling losses in this study (n = 7) may be unrepresentative of sibling loss in general.

There is extensive recent literature on the psychological status of adult survivors of childhood cancer, but less data is available specifically on the psychological status of AeYA oncology patients. Survivors of childhood cancers who received the diagnosis of cancer as AeYAs experience greater psychological distress than do survivors who received the diagnosis of cancer earlier in childhood, and those who undergo more intense cancer treatment have poorer psychosocial outcomes when compared with patients who receive less intense therapy[30,31]. Compared to sibling controls, AeYA survivors of cancer whose disease was diagnosed when they were between 11 and 21 years of age self-report higher levels of anxiety, depression, and somatization[21]. The Swiss Childhood Cancer Survivor Study also found survivors who were older than 10 years at diagnosis to be at greater risk of emotional distress when compared to adults[31]. Michel et al posit that adolescents fully understand the life-threatening[32,33] aspects of their disease at a time when they are attempting to achieve the developmental norms of their age. The risk factors for adverse psychological outcomes in AeYA patients with cancer or in recent survivors have not been well described. Although the implications of losing loved ones or peers while coping with malignant disease have never been quantified, such loss may be one risk factor for negative mental health outcomes.

Because the effect of parental bereavement has been described elsewhere in the literature and parentally bereaved AeYAs represent only a minority of our study population, we will focus the remainder of our discussion on peer loss, the second most common type of loss experienced by AeYAs with illness. Peer loss is a durable event; after the loss of a classmate to cancer, 89% of students reported experiencing permanent change, thinking and worrying more about dying, and a significant number of classmates had continued grief symptoms 18 months after the death[34,35]. Another study found continued grief reactions and symptoms of posttraumatic distress 9 months after the sudden death of a peer: 38% of responders reported that they would never overcome the loss, whereas 8% believed it would take years for them to do so[36]. Adolescents who have experienced another death in addition to peer loss indicate the loss of a friend to be “qualitatively different”[37]. Teenagers believe they need 1 to 3 months of ongoing support from parents or other peers after experiencing a peer loss, whereas they desire only “a few weeks” after a grandparent loss, indicating a need for additional support for AeYAs in coping with peer loss[1,26].

The perceived emotional closeness between the mourner and the deceased is a mediator of bereavement; thus, a greater degree of perceived familiarity can often result in more intense grief[37]. Because adolescents frequently report a greater closeness with their friends than with their grandparents, they commonly experience more intense grief after the loss of a friend than after the loss of a grandparent[19]. The more a teen identifies with the deceased, the more likely they are to consider their own mortality[38]. In a high school study on peer loss, the frequency of contact and perceived closeness were highly correlated; greater closeness was associated with more difficulty in accepting the loss[36]. Difficulty with peer loss appears to stem from the normative belief that peers are too young to die and from the loss of a security-enhancing peer relationship[39]. Unlike younger children, AeYAs have the cognitive capacity to understand the permanence and implications of death and to relate to the death of a peer. Because of their shared experiences, AeYA patients probably feel similar to other children with illness, and the contact they have during or immediately after treatment may result in a high degree of perceived closeness that can make AeYAs with illness particularly vulnerable to loss. Difficulty with peer loss has been described by AeYA survivors; one study participant publicly noted the following:

…a fellow patient named Carissa passed away today. Feeling this way, feeling so tied to death through love, takes me right back to losing Odie [another patient] a few months ago, and all I can do is wonder, “Why?” “Why this girl? Why this little boy? Why did they get types of pediatric cancer that don’t respond well to chemo?” And then the biggest question of all: “Why am I alive when they aren’t?”[40]

Adolescents are often disenfranchised grievers without a socially defined, normative place or way to grieve, placing them at increased risk of emotional complications[1]. Adults often fail to recognize peer loss or its effects, and teenagers frequently report disappointment in parental response, feeling discomfort in talking about feelings with them[39]. AeYAs desire more social support than they actually receive for most losses, particularly disenfranchised peer losses[1]; further research on social support following loss would appear to be beneficial for the design of effective support services for AeYAs. AeYAs rarely talk about an important loss, even with their close friends or parents. This is concerning, given that two-thirds of AeYAs reported that their bereavement was slow or that they never got over the loss. The ability to derive meaning from the loss of a loved one is fundamental to a healthy grief process[38]. The private, often unrecognized grief experienced by adolescents may make it more difficult for AeYAs to talk about and process (i.e., make meaning of) a death[38]. It was surprising to find no relation between loss importance and psychological symptoms. Controlling for time since loss may provide additional insight into the negative impact of loss.

Exposure to multiple losses appears to be more common in our population of AeYAs with cancer (being reported by 61%) than in the general population of AeYA (of whom 40% reported multiple losses)[1]. This indicates that further research on AeYA grief and bereavement is necessary, particularly in the setting of chronic or life-threatening illness (cancer, cystic fibrosis, muscular dystrophy, etc.). Our research found associations between negative psychological outcomes and peer loss, but further research is warranted to explore the risk of somatization and the trend toward depression in AeYA with illness who experience peer loss. Because of the significant effect that an important loss may have on an AeYA, screening for unrecognized grief symptoms such as somatization or death anxiety may be warranted. A simple Likert scale can be used to identify AeYAs who have experienced a loss of high importance (with a rating of 9 or 10), and these patients can be screened for complications in processing their grief. Pediatric hospitals may seek to include grief and bereavement support within the medical home. It may be appropriate to support AeYAs at an individual level by promoting access to counseling or by drawing upon institutional resources (chaplaincy, child life, social work, psychology) to develop programmatic-level interventions such as peer support groups, music therapy interventions, or other bereavement-focused group interventions.

This study sought to quantify the epidemiology of loss and to describe the bereavement experiences of AeYAs with cancer while exploring the relation between loss and known psychosocial correlates. Future research might follow bereaved AeYAs longitudinally to elucidate (1) the impact of time since loss on the bereavement experience of AeYAs and (2) the longitudinal impact of loss, and possibly multiple losses, over time. The present study is limited by its cross-sectional design based on data collection from AeYAs at a single institution and may not reflect the experiences of patients seen in other settings. Individuals were often unable to remember the exact month and year of their loss, which prevented an analysis of time since loss and is an important study limitation. The age of the deceased was not collected on the bereavement questionnaire, which may be a limitation; some respondents verbally commented to study staff that the death of younger children seemed “unfair,” but we were unable to analyze the impact of the age of other deceased children on AeYA bereavement. Finally, it is unknown whether these findings are generalizable to other AeYA patients with serious illness. It will be important to examine the relation between peer loss and the participant’s own illness trajectory, particularly when the deceased and participant share the same diagnosis (i.e., cancer). Formal assessments of grief with validated grief inventories would be beneficial, and research on the bereavement experiences of healthy AeYAs or AeYAs with other chronic illnesses would be informative in identifying whether the pediatric oncology experience is different from that of other AeYA populations.

## Conclusions

This study indicates that peer loss is more common for AeYAs than in the general population. Given that numerous studies have demonstrated the unique nature of peer loss, clinical assessments for loss and bereavement difficulties appear to be important in the population of AeYA patients with a serious illness such as cancer. Parental and peer loss is associated with the risk of negative mental health outcomes in AeYA patients with cancer and warrants further exploration. Longitudinal research on the grief and bereavement experiences of AeYAs with illness is urgently needed. Research on clinical interventions targeting grief in AeYAs with cancer would also be beneficial.

## Supporting information

S1 File Sample of bereavement questionnaire. (DOCX)

S1 Table Manner of death for non-pet losses. (DOCX)

S1 Dataset ALOSS manuscript data. (XLSX)

S2 Dataset ALOSS manuscript data dictionary. (XLSX)
