# Supplementary material for: The bereavement experience of adolescents and early young adults with cancer: Peer and parental loss due to death is associated with increased risk of adverse psychological outcomes
Source: PLoS One. 2017 Aug 23;12(8):e0181024. doi: 10.1371/journal.pone.0181024 (PMC5568383; doi:10.1371/journal.pone.0181024)
Supplement: S1 File — (DOCX) [file pone.0181024.s001.docx]

**Sample of Bereavement Questionnaire:**

***I HAVE LOST THROUGH DEATH*:**

| ***Example*-**  **Deceased Loved Ones** | **Yes** | **If Yes, year of death** | **Manner of death (Violent-accident, homicide, suicide; Natural-expected—for example, died after a long illness; Natural –unexpected—died suddenly of natural causes, for example, a heart attack)** |
| --- | --- | --- | --- |
| Grandmother | X | ? / don’t know | Natural-expected |
| Friend ** | X | 2011 | Unexpected - cancer |
| Treasured pet | X | Dog—2010 | Violent—hit by a car |

| **Deceased Loved Ones** | **Yes** | **Year of death**  **(if known)** | **Manner of death*** | **Did you attend**  **a wake, funeral, or**  **Memorial Service** | | | | | **Mark if death**  **was**  **cancer related** | |
| --- | --- | --- | --- | --- | --- | --- | --- | --- | --- | --- |
| **Mother** |  |  |  |  | | | | | |  |
| **Father** |  |  |  |  | | | | | |  |
| **Stepmother** |  |  |  |  | | | | | |  |
| **Stepfather** |  |  |  |  | | | | | |  |
| **Brother** |  |  |  |  | | | | | |  |
|  |  |  |  |  | | | | | |  |
| **Sister** |  |  |  |  | | | | | |  |
|  |  |  |  |  | | | | | |  |
| **Grandmother (Mom’s)** |  |  |  |  | | | | | |  |
| **Grandmother (Dad’s)** |  |  |  |  | | | | | |  |
| **Grandfather (Mom’s)** |  |  |  |  | | | | | |  |
| **Grandfather (Dad’s)** |  |  |  |  | | | | | |  |
| **Aunt** |  |  |  |  | | | | | |  |
|  |  |  |  |  | | | | | |  |
| **Uncle** |  |  |  |  | | | | | |  |
|  |  |  |  |  | | | | | |  |
| **Close friend, classmate, fellow patient** (use initials) |  |  | *Manner of death* | *Attended services* | *Cancer related death?* | | | *Type of cancer, if known* | | |
| **1** |  |  |  |  | Yes | No | |  | | |
| **2** |  |  |  |  | Yes | No | |  | | |
| **3** |  |  |  |  | Yes | No | |  | | |
|  |  |  |  |  | Yes | No |  | | | |
| **Other loved one** |  |  |  |  |  | |  | | | |
|  |  |  |  |  |  | |  | | | |
| **Treasured Pets** |  |  |  | ***CIRCLE* or *STAR* the MOST IMPORTANT LOSS in your life.** | | | | | | |

**If none of the losses were very important circle: N/A**

(STOP HERE – if you had no losses on the previous page)

**CIRCLE how important (significant) to you was this loss?**

**1 2 3 4 5 6 7 8 9 10**

(Not at all important) (Neutral) (Very Important)

**Since the death(s) I have talked about the loss(es):**

*Always Often Sometimes Rarely Never*

**The loss(es) changed my life:**

*Enormously A lot A little It didn’t change my life much*

**I got over the loss(es):**

*Right away Quickly Slowly I never got over it*

**I felt I needed more professional help:**

*Always Often Sometimes Rarely Never*

**Since the death(s) I have talked about it with:**

| **My parents** | ***Always*** | ***Often*** | ***Sometimes*** | ***Rarely*** | ***Never*** |  |
| --- | --- | --- | --- | --- | --- | --- |
| **Other adults in my family** | ***Always*** | ***Often*** | ***Sometimes*** | ***Rarely*** | ***Never*** | ***n/a*** |
| **My brother(s) or sisters (s)** | ***Always*** | ***Often*** | ***Sometimes*** | ***Rarely*** | ***Never*** | ***n/a*** |
| **My friends** | ***Always*** | ***Often*** | ***Sometimes*** | ***Rarely*** | ***Never*** |  |
| **A school counselor** | ***Always*** | ***Often*** | ***Sometimes*** | ***Rarely*** | ***Never*** |  |
| **My doctor or nurse** | ***Always*** | ***Often*** | ***Sometimes*** | ***Rarely*** | ***Never*** |  |
| **On-line (social networking site, chat groups, on-line support groups)** | ***Always*** | ***Often*** | ***Sometimes*** | ***Rarely*** | ***Never*** | ***n/a*** |
| **Psychologist/ therapist / psychiatrist** | ***Always*** | ***Often*** | ***Sometimes*** | ***Rarely*** | ***Never*** |  |
| **A bereavement counselor, child life specialist, chaplain or social worker** | ***Always*** | ***Often*** | ***Sometimes*** | ***Rarely*** | ***Never*** |  |
