# Supplementary material for: The bereavement experience of adolescents and early young adults with cancer: Peer and parental loss due to death is associated with increased risk of adverse psychological outcomes
Source: PLoS One. 2017 Aug 23;12(8):e0181024. doi: 10.1371/journal.pone.0181024 (PMC5568383; doi:10.1371/journal.pone.0181024)
Supplement: S1 Table — (DOCX) [file pone.0181024.s002.docx]

**S2: Supplemental Table: Manner of death for non-pet losses**

| **Manner of death** | **Type of loss** | | | | | | | | | | | |
| --- | --- | --- | --- | --- | --- | --- | --- | --- | --- | --- | --- | --- |
|  | **Sibling(s)** | | **Parent(s)** | | **Grandparent(s)** | | **Friend(s)** | | **2nd degree relative(s)** | | **Other loved one(s)** | |
|  | **n** | **%** | **n** | **%** | **n** | **%** | **n** | **%** | **n** | **%** | **n** | **%** |
| *No. of with loss* | *7* |  | *8* |  | *89* |  | *56* |  | *52* |  | *8* |  |
| *Total no. of losses* | *8* |  | *11* |  | *153* |  | *95* |  | *74* |  | *8* |  |
| Cancer | 0 | 0 | 1 | 9 | 42 | 27 | 63 | 66 | 23 | 31 | 4 | 50 |
| Natural-expected | 1 | 13 | 0 | 0 | 25 | 16 | 0 | 0 | 3 | 4 | 1 | 13 |
| Natural-unexpected | 4 | 50 | 4 | 36 | 42 | 27 | 2 | 2 | 20 | 27 | 2 | 25 |
| Natural-unknown | 0 | 0 | 0 | 0 | 23 | 15 | 3 | 3 | 3 | 4 | 0 | 0 |
| Traumatic-accidental | 2 | 25 | 6 | 55 | 5 | 3 | 18 | 19 | 12 | 16 | 1 | 13 |
| Traumatic-violent | 0 | 0 | 0 | 0 | 0 | 0 | 8 | 8 | 6 | 8 | 0 | 0 |
| Unknown | 1 | 13 | 0 | 0 | 16 | 10 | 1 | 1 | 7 | 9 | 0 | 0 |
